# Present state of reproductive medicine in Japan – ethical issues with a focus on those seen in court cases

**DOI:** 10.1186/1472-6939-7-3

**Published:** 2006-04-05

**Authors:** Mayumi Mayeda

**Affiliations:** 1 Department of Biomedical Ethics, School of Health Science and Nursing, The University of Tokyo Graduate School of Medicine, 7-3-1 Hongo, Bunkyo-ku, Tokyo 113-0033, Japan

## Abstract

**Background:**

Against a background of on the one hand, a declining demography and a conservative family register system that emphasizes the importance of the blood line, and on the other hand, an increase in the number of people undergoing fertility treatment, the absence of a legal regulatory framework concerning ART matters is likely to result in an increasing number of contradictory situations. It is against this background that the paper sets out to examine the judgements of court cases related to ART, with a particular focus on the legal determination of parental status, and to link these to aspects of the legal and socio-ethical environment within which the courts make their judgements.

**Methods:**

The methods used were thorough investigation of all the court cases concerning ART in the public domain in Japan, including the arguments of the concerned parties and the judgements so far delivered. With the court cases as a central focal point, trends in Japan, including deliberations by government and academic societies, are reviewed, and the findings of surveys on the degree of understanding and attitudes among the people toward ART are summarized.

**Results:**

In terms of the judgements to date, the central criteria used by the courts in determining parental status were the act of parturition and the consent of the husband of the concerned couple. The government and academic societies have displayed a cautious attitude toward ART, but the findings of attitude surveys among the people at large show a generally positive attitude toward ART. Attitudes toward the overwhelming importance hitherto attached to the bloodline are also seen to be changing.

**Conclusion:**

The main conclusion is that in the absence of a legal regulatory framework for ART, there is likely to be an increase in the contradictions between the use of outdated legal precedents and the technical development of ART. Since much of the specialist discussion necessary for the formulation of a legal framework has already been carried out, the speedy enactment of comprehensive and at the same time flexible legislation would be highly desirable, but further wide-ranging discussion involving the general public is likely to be needed first.

## Background

The core issue explored in this paper, namely the determination of parental status in the case of children born with the help of ART, must be located within ART as a whole, and this in turn must be seen against the background of Japan's demographic situation. For several years past, the birth rate in Japan has been declining, and in 2005, Japan's total population began to decline. The Japanese government has announced a package of measures designed to stem the falling birth rate and, although ART is not specifically included in them, it is likely that widespread awareness of the demographic situation will contribute to increased understanding of, and toleration of, ART.

A further important constituent element of the general background situation is the Japanese family register system. Reference is made at several points in the paper to the importance of blood ties and the blood line in the eyes of Japanese people. The family register system is the specific manifestation of this importance. Introduced at the beginning of Japan's modern era in 1872, the family register, a composite record of births, marriages, deaths and changes of residence, marks the evolution of a family over many generations. It takes the place of individual birth certificates used in many Western countries, and shows the importance attached to the blood line. Voices calling for modification of the system have been gradually increasing for several years past, but at present, such voices are still countered by more conservative voices claiming that any modification would lead to the destruction of Japan's traditional family values.

Turning to more specific factors, it was on July 16, 2004, that the decision of a court on the legal status of a baby born by in vitro fertilization (IVF) was handed down in Japan for the first time. In this case, a plaintiff claimed that a baby born by IVF using the frozen sperm of her late husband should be legally recognized as his child. Takamatsu District Court dismissed this claim, on the grounds that "the social perception that a baby born in such way is a child of the dead husband is not sufficiently strong". This judgment serves as an illustration of the point made in the preceding paragraph.

It is estimated that there are approximately 284,000 patients who are undergoing fertility treatment in Japan, and the number of babies born by assisted reproduction technologies (ART) has been put at 59,520. However, a regulatory framework of law and guidelines concerning ART is virtually non-existent. The fact is that, effectively, ART is governed by the guidelines set out in reports issued by specialist committees, explored later in the paper, and by voluntary rules established by doctors and their professional societies.

Despite, or perhaps because of, this kind of situation, there are fewer legal conflicts concerning ART in Japan than in other advanced nations. As explanations for this, one could cite the relatively low level of social awareness of ART to date due to the emphasis put on the concept of the bloodline as explained above, and the fact that the rate of expansion of ART has been slow. Recently, however, in Japan too, the various issues concerning ART, with particular focus on the determination of parental status, have gradually come to the surface, and judicial settlement of the issues has become increasingly a matter of concern. Factors such as developments in medical technology, for example, the use of frozen sperm, as well as changes in social trends due to wider recognition of ART are likely to lead to an increase in the number of difficult problems faced by the courts due to the gulf between the expansion of ART on the one hand and the delay in introducing a legal regulatory framework for ART on the other.

With a central focus on court cases, the paper examines the issues involved in the determination of parental status with reference to the various individual patterns of ART, and gives an overview of the results of reports and surveys as well as the main socio-ethical issues that constitute the background against which the judgments of the courts are formulated and delivered.

## Methods

### Organization of ART patterns

The paper classifies the various patterns of ART in terms of the modalities of conception and gestation, listed at the start of the Results section and in Additional file 2.

### Court cases concerning ART in Japan

Taking the classification as a reference point, the paper explores the legal determination of parental status and the legal status of the children born with the help of ART through an examination of those cases concerning ART that have come to court in Japan. In every such case, the paper investigates the allegations of all the parties concerned and the judgment of the courts.

### Trends in Japan

With a view to elucidating the wider background against which judgments are made, the paper reviews and discusses trends in Japan concerning ART, including deliberations on the part of the government and the opinions of academic societies.

### Attitudes of citizens toward ART

With a view to ascertaining the degree of understanding and permeation of conscious awareness of ART among the Japanese people as well as their attitudes toward ART, the paper refers to the results of a survey conducted with the help of a Government grant in 2003. In this survey, approximately 8,000 subjects were randomly sampled so as to investigate the attitudes toward ART, and the collection rate was more than 60%.

## Results

### Organization of ART patterns

Using the modalities of conception and gestation as criteria, it is possible to distinguish broadly 9 patterns of ART (listed in Additional file 2) as follows:

I) AIH;

II) AID;

III) IVF using the sperm and eggs from a concerned couple;

IV) IVF using donor sperm and eggs from the wife of a concerned couple;

V) IVF using sperm from the husband of a concerned couple and donor eggs;

VI) IVF using donor embryos;

VII) using the embryos of a concerned couple in a borrowed womb;

VIII) using the husband's sperm and donor eggs in a borrowed womb (surrogacy);

IX) surrogacy using donor sperm and donor eggs.

**Figure 1:**
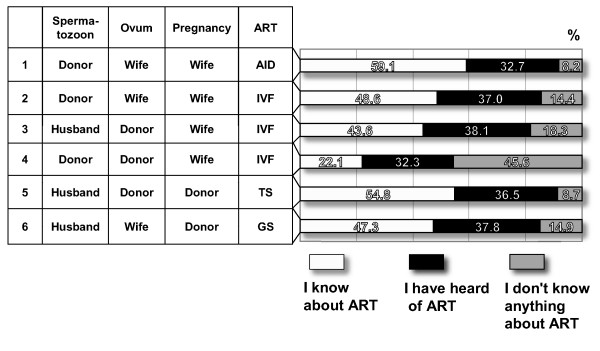
**Social penetration of knowledge about ART**. This figure shows the penetration ratio of knowledge about ART in Japan. Approximately 50% of subjects knew about AID, IVF (donor sperms and donor eggs), and surrogacy.

**Figure 2:**
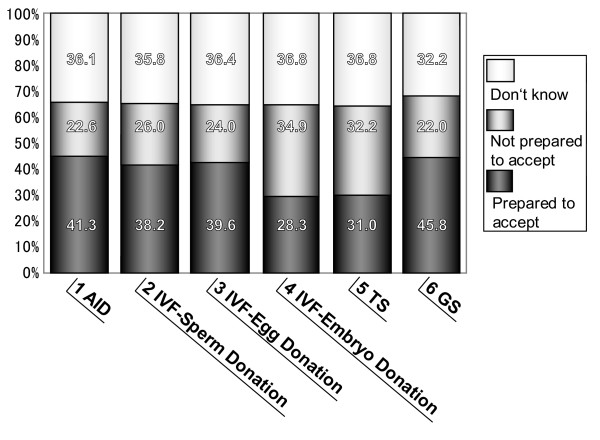
**The national consciousness regarding "social preparedness to accept ART"**. This figure shows the question as to whether each type of ART should be socially accepted. Most subjects accepted forms of ART, including surrogacy, where the sperm and eggs were those of the concerned couple.

The following commentary on the above 9 patterns of AID provides an overview of the potential and actual problems associated with each pattern. Reference is made in respect of the various patterns to the degree of penetration of knowledge about ART and to the degree of acceptance. The data is tabulated in Figures 1 and 2. The source of the data is "Research on Attitudes of Citizens toward Assisted Reproductive Technology", issued as a report on a Scientific Special Research project carried out with the help of a Grant in Aid for Scientific Research from the Ministry of Health, Labour and Welfare in 2003 [1]. Further details of this research can be found under the heading "Attitudes of citizens toward ART" later in this Section. In the case of those patterns that have already featured in court cases, a brief mention is made of this, and the reader is referred for further details to the text under the heading of Court Cases later in this Section.

#### I) AIH

AIH is the most commonly performed form of ART. Sperm and eggs from the concerned couple are used, and the wife herself gives birth. The resulting child is, of course, their legitimate child. Knowledge of AIH has penetrated socially to 70% of the population. It is often carried out as the first step in treatments for sterility of an uncertain cause. AIH is not covered by health insurance, and costs about 20,000 yen (around 175 US dollars). As problems concerned with AIH, one could cite the usage of the husband's sperm without his consent and the usage of frozen sperm after the husband's death, but there are as yet no legal precedents for cases of this kind.

#### II) AID

Due to the use of donor sperm in AID, the relationship between the resulting children and their father can become problematic. In such cases, according to judicial precedents, the resulting child is regarded as a legitimate child when the consent of husband is confirmed by a "clear indication of intention" or a "written form", and denial of the legitimate child at a later date is not allowed, using the concepts of "betrayal" and "the importance of results" as criteria. Most of the problems concerning AID are linked to the father-child relationship, for example, "custody claim" and "denial of legitimacy". This pattern of ART has featured in 2 court cases, discussed as case i) and case ii) under "Court cases". According to the research on the attitude of citizens (hereafter, "the research"), the social penetration ratio of knowledge of AID is around 60% (see Figure 1), and 40% accept AID (see Figure 2). The history of sperm donation in Japan dates back more than 50 years, and there is no particular problem in regarding a resulting child as a biological child if the wife gives birth and the husband "agrees" to AID. For further details, please see "Court cases" later in this section, and the discussion of "Court cases" in the Discussion section.

#### III) IVF using sperm and eggs from a concerned couple

There are no major problems with this pattern of ART since both the sperm and eggs used are those of the concerned couple, and it is the wife of the concerned couple who gives birth. This method is similar to AIH, but the mechanical action of in vitro fertilization intervenes. Judicial precedents of IVF concerning the parent-child relationship comprise only a case of posthumous recognition (see court case iii)), and the criteria used to determine the judgment were "consent of the father" and the "existence of a blood relationship". The judgment of the Supreme Court is still awaited. Potential problems with this kind of IVF are likely to be concerned with posthumous recognition of, and determination of the status of, a child born by the use of the husband's sperm after his death as in the quoted case. According to the research, approximately 50% (see Figure 1) of people are knowledgeable about, and 90% have heard something about, this pattern of ART. This is one of the most commonly performed fertilization procedures as the step subsequent to AIH (pattern I). Recognition that a child born as a result of this procedure is a legitimate child of the concerned couple is not in itself problematic, and does not spark much social criticism.

#### IV) IVF using donor sperm and eggs from a wife and V) IVF using sperm from a husband and donor eggs

In these types of IVF, the wife of a concerned couple gives birth to a child, and either the husband or the wife of the concerned couple has a genetic relationship with the child. As with III above, recognition of a resulting child as a legitimate child of the concerned couple is not problematic provided that there is "consent" and the "existence of a blood relationship" with either member of the concerned couple. According to the research, approximately 50% of people are knowledgeable about, and 80% have heard something about, these types of IVF. Regarding the parent-child relationship, 60% agree that children born by these types of IVF should be regarded as legitimate children of the couple. The Government sets rules for cases of IVF using donor sperms or eggs, and the Japan Society of Obstetrics and Gynaecology identifies sperm donation ethically with AID [2]. Problems that can occur with these types of IVF include the legal status of children born by IVF without consent

#### VI) IVF using donor embryos

In the case of this pattern of ART, there are some obstacles in the way of regarding a resulting child as the legitimate child of the concerned couple, since donor embryos are used and there is no genetic relationship between the couple and the child although the wife gives birth. In terms of the procedures used, this method is similar to that of a person who makes a contract as a surrogate mother, gives birth and ends up being acknowledged as he mother of the resulting child. The requirement of "consent", which is a feature of judicial precedents, is satisfied, but the problem of how the "genetic" relationship should be viewed remains to be solved. The government had until now allowed the usage of embryos with some conditions attached, but the Japan Society of Obstetrics and Gynaecology does not approve of it, since the parent-child relationship can become complicated. The resulting child will have two different couples as his/her parents (i.e., the biological parents, and a birth mother and social father). According to the research, approximately 50% know about or have heard about IVF using donor embryos, and only 30% accept it. Concerning the parent-child relationship, 40% agree to the recognition of the resulting child as the legitimate child of the concerned couple, but another 40% answer that they do not know. There is clearly scope for wide disagreement on the issue of the determination of legal parental status in the case of this pattern of ART, but as yet, there are no legal precedents.

#### VII) Borrowed womb using the embryo of a concerned couple

Under this procedure, a third person gives birth using the embryo of a concerned couple. A couple, who worked as entertainers in Japan, used surrogacy abroad, and the birth registration of the resulting child was rejected. This case aroused discussion in Japan. In this case, the claim was rejected because the "mother did not actually deliver the child", based on the judicial precedents that "the legal mother-child relationship should be accepted only between a person who delivers a child and the child that is delivered", and "the resulting child should be regarded as an adopted child". The key problem, as with other kinds of surrogacy, would seem to be the degree of importance to be attached to the act of parturition in determining legal parental status. According to the research, the penetration rate of knowledge about surrogacy has reached 50%, and about 50% of subjects accept the concept of a borrowed womb. In addition, 60% agree to the recognition of the resulting child as the legitimate child of the concerned couple.

#### VIII) Surrogacy using the husband's sperm and donor eggs

In this pattern of ART, when a surrogate mother gives birth to a child by using her own eggs and the sperm of the husband of the concerned couple, there is no problem about recognizing the genetic link between the husband and the resulting child. However, there are some problems on the wife's side since there is "no fact of childbearing" and "no genetic relationship with the child", although there is "consent" to surrogacy. The child is born under a surrogate-motherhood contract. However, when looked at objectively, this situation is very similar to that that in which a wife recognizes and takes in as her own a child born between her husband and another female. In terms of judicial precedents, the wife who did not actually deliver the child should be considered as the mother of the child by adoption. The government also emphasizes the idea that "the person giving birth is the mother", and regards the husband as the father and the surrogate mother as the mother. According to the research, 50% of subjects know about this type of surrogacy, and approximately 30% accept it. Approximately 50% agree to the recognition of the resulting child as the biological child of the concerned couple. As discussed under VII above, the key point is the priority to be given to the act of parturition in determining legal parental status. The only court case in Japan to date related to this pattern of ART was concerned with birth registration (see details under Court case iv)), but it serves as an illustration of the key point referred to in the previous sentence. Other problems that can occur in the case of surrogacy include custody battles between the concerned couple and a surrogate mother, what to do when the resulting child is born with a handicap, and one-sided dissolution of a contract by a surrogate mother during pregnancy. Such cases have not as yet been reported in Japan.

#### IX) Surrogacy using donor sperm and donor eggs

In this case, a surrogate mother gives birth by using an embryo from a third person, and the concerned couple is not involved at all. This case is almost identical to a simple adoption, in which a couple takes in a child from a surrogate mother. The only difference is that the adoption depends on a surrogate-motherhood contract with the concerned couple. The most desirable procedure is for the couple to take in a child by general adoption, and the necessity of this surrogate-motherhood contract is very questionable. There would seem to be no problems in treating this case as a case of general adoption.

### Court cases concerning ART in Japan (Additional file 1)

The above section provides an overview of the different patterns of ART and outlines the kind of legal problems that can arise in respect of each pattern. This section examines the cases that have actually come to court.

#### i) – An issue of who the person in parental authority should be in a case of artificial insemination with donor's semen (Pattern II: AID)

##### Background

Y (father) and X (mother) got married on November 22, 1990. With the agreement of Y (who was sterile) and X, X received donor sperm and delivered Z (a boy) by artificial insemination on February 6, 1994. Y and X lived separately from March 9, 1996, and Z was nurtured alternately by Y and X. On January 22, 1997, Y and X obtained a divorce through the Family Court, and waged a custody battle.

The original judgement [3] handed down by Niigata Family Court on March 30, 1998, gave the custody of Z to Y for the following reasons. "Although Y is not the biological father of Z, the donor of the sperm has not been identified, AID was performed with the agreement of X and Y, and Z is a legitimate child. In addition, X and Y are each qualified to be a person in parental authority. With these points as preconditions, Z looks more stable mentally at Y's house where he has continuously lived till now." X immediately appealed.

##### Judgement

Tokyo High Court gave the custody of Z to X on September 16, 1998 [4], for the following reasons. "To determine who should be the person in parental authority for the minor, Z, it is necessary to take into consideration the fact that there is no blood relationship between Y and Z. At the same time, however, it is not the case that the mother should necessarily be designated as the person in parental authority. Basically, given the need for comprehensive consideration from the viewpoint of Z's well-being and other factors, in the light of Z's age, it is judged to be reasonable that X should be the person to be given parental authority over Z." The judgment of the Tokyo High Court was not appealed to the Supreme Court. The case is discussed in greater detail in the Discussion Section, but it is interesting that in the case of the family court and the high court judgements, although the two courts reached different conclusions, the presumed welfare of the child seems to have been made the primary consideration.

#### ii) – An issue of denial of legitimacy in an AID case (ART Pattern II)

##### Background

Y (father) and X (mother) got married on March 31, 1992. Since Y and X were unable to have a child, fertility treatment had been continuing since 1993. X became pregnant once by artificial insemination with her husband's semen (AIH), but had a miscarriage. At the end of 1994, Y and X lived in a state that in practical terms amounted to divorce, but without divorce papers being filed and consequently without X's name being removed from Y's family register. In this situation, X became pregnant through AID and delivered the baby Z (a girl) on January 27, 1997. Y named Z and registered the birth of a legitimate child. Subsequently, Y raised doubts about Z's birth of, and filed a complaint for denial of legitimacy due to X's adultery. X contested the issue, claiming that Y approved AID in advance, and recognized Z as a legitimate child.

##### Judgement

Osaka District Court accepted the claim of Y on December 18, 1998 [5], for the following reasons. "Y insisted that X engaged in adultery, but this could not be confirmed in the absence of supporting evidence. At the same time, however, X's assertion that Y had given advance approval to AID was not acceptable since X and Y did not prepare a letter of consent to AID in advance. Giving the child a name and filing notice of the birth could not in themselves be said to indicate Y's intention to recognize a lawful child."

The case, which is discussed more fully in the Discussions Section, was not appealed to the High Court. The key point that determined the court's thinking in this case was the absence of any written evidence that Y had agreed to AID, as opposed to AIH, in the context of fertility treatment.

#### iii) – An issue of posthumous recognition in an IVF case (ART Pattern III)

##### Background

Y (father) and X (mother) got married in 1997. Y had suffered from chronic myelocytic leukemia since 1990, and had been continuously treated. After the marriage, both Y and X continued to receive fertility treatment. Six months after the marriage, Y underwent bone marrow transplantation. Since it was probable that Y would develop aspermia due to a side effect of the treatment, Y's sperm was frozen and put into storage before the transplantation. Y died in 1999. X received IVF by using Y's sperm, and delivered Z in the summer of 2001.

In 2002, X tried to have the birth of Z registered as a legitimate child of Y and X, but the application was not accepted since Article 772–2 of Civil Code prescribes that "A child born 300 days or more after the day on which the marriage was dissolved, shall not be accepted as a lawful child". X did not accept the ruling and appealed to the Family Court [6], but the claim was rejected on December 20, 2001, for the following reasons. "A legitimate child shall be the child conceived or born between a man and a woman in a marital relationship. A child that is conceived or born after the marriage was dissolved due to the death of the father, is not a legitimate child." X immediately appealed, but Takamatsu High Court rejected the appeal, "Z could not be presumed to be a legitimate child as defined in Article 772 of Civil Code, nor could she be seen as a legitimate child on the grounds of her conception, since this took place after the marriage was dissolved due to the death of the father [7]". X filed a special appeal, but it was also rejected.

After the special appeal of the mother was finally discharged, the mother brought a separate action on behalf of the child as her *prochain ami*, seeking recognition of Z. In the first trial, Matsuyama District Court dismissed this claim, because "it was not clear that Y agreed to IVF" and "there is still insufficient social consensus that a baby born in such way should be seen as a child of the dead husband" [8,9]. In response to this, X insisted that "Y did agree to IVF", and argued that "if the Civil Law cannot make presumptions that cover this case, the inadequacy of the law should be compensated for by the Constitution".

##### Judgement

Takamatsu High Court accepted X's claim on July 16, 2004 [10]. The court presented for the first time criteria that could form a basis for judgement when it said that "it is sufficient that there are natural blood relations between Y and Z and that the consent of the father was given, and there are no grounds for requiring that the physical existence of the father at the time of the mother's pregnancy should be made a condition for recognizing the legitimacy of the child". However, the government objected to this judgement and filed an appeal with the Supreme Court on July 29, saying that "since there are no judicial precedents, we would like to leave a decision in this case to the judgement of the Supreme Court".

At the time of writing this paper (January 2006), the Supreme Court judgement is still outstanding. The key point in the High Court proceedings is that the court felt that the consent of the father and the existence of a blood relationship between the father and the child were sufficient to override the time limit specified in the Civil Code. The case is discussed further in the Discussions section.

#### iv) An issue of birth registration of a child delivered by a surrogate mother (ART pattern VIII)

##### Background

A couple in their 50s, living in the Kansai District of Japan, were receiving ongoing fertility treatment. An egg donated by an Asian American woman was fertilized in vitro with the husband's sperm, after which the fertilized egg was implanted in another American woman, who acted as a surrogate mother and gave birth to twins in October 2002. A U.S. birth certificate was issued, but when the couple tried to have the birth of the twins registered at the Japanese Consulate General in the U.S., the Japanese Ministry of Justice decided to reject the application in November 2003 on the basis of the judicial precedent of the Supreme Court in 1962 [11] that "a person who delivers a child shall be the mother". Subsequently, the couple tried to have the birth registered at their local government office in Japan in January 2004, but their submission was also rejected in February 2004. The couple appealed the unacceptable rulings to the Family Court demanding reversal of the rejection.

##### Judgement

The Akashi Branch of the Kobe Family Court decided to reject their claims on August 14, 2004, for the following reasons. The wife is neither the person who provided the egg nor the person who delivered the twins, and "seen from the perspectives of objectivity and precision, the legal mother-child relationship should be accepted only between a person who delivers a child and the child that is delivered", and this issue "should be handled by adoption". The couple immediately appealed to Osaka High Court on August 24, 2004, emphasizing that "we find it highly regrettable that even though a birth certificate was issued in America, the birth registration is not accepted in Japan", and that "judicial precedent of the Supreme Court, delivered at a time when current advances in the field of assisted reproductive technology (ART) were still unknown, to be applied to our case." According to a press report, the Osaka High Court delivered a judgment on May 20, 2005, dismissing the claim. The Court said that "an act of parturition is necessary to establish a mother-child relationship, and even if medical technology has developed, that is not a reason to recognize this case as an exception". Thereupon, the couple appealed to the Supreme Court, but again according to a press report, the Supreme Court also rejected the appeal, ruling that the case should be settled by adoption. Details of these judgments are not included in Additional file 1, because no formal report has yet been issued.

In this case, therefore, which is primarily focused on the issue of birth registration, the courts have followed the view, traditional in Japan, that the act of parturition should be the primary criterion in determining legal parental status. The case is discussed further in the Discussions Section.

### Trends in Japan

It may be usual for improvements in legislation in response to progress in medical science to suffer from delays. However, in the case of high-state-of-the-art medical technology that has the potential to shake the foundations of the human race, there is an urgent need for a globally consolidated response. Currently, the development of legislation concerned with ART is underlain by Regulations relating to Human Cloning Techniques and Other Similar Techniques (December 2000) [12] and Guidelines for ES Cells [13]; and that concerned with AID and IVF rests on the foundation of the guidelines and reports issued by academic societies, reports from specialist committees, and individual self-restraint established by doctors. A legal framework has not yet been established, and deliberations at government level are still continuing. The following paragraphs outline the developments to date in terms of governmental responses and the climate of public opinion with a view to setting out the background against which the decisions of the courts are delivered.

#### i) Governmental deliberations on ART in Japan

Deliberations on ART at government level in Japan began in October 1998, when a high-level specialist committee was set up under the auspices of the Health Science Council with the aim of considering the issues associated with ART from a broad perspective. The committee submitted a "Report on Assisted Reproductive Technologies using Donor Sperm, Eggs and Embryos" on December 28, 2000. On June 11, 2001, with the aim of carrying out a further investigation of ART using donor sperm, eggs and embryos, the Assisted Reproductive Technology Committee was set up, also under the auspices of the Health Science Council. In parallel with these developments on the health and medical side, the ART-related Parent and Child Jurisprudence Sub-Committee was established on February 16, 2001 under the auspices of the Legislative Council, and its deliberations are continuing.

With a view to consolidating the reports and deliberations emerging from these initiatives, the Assisted Reproductive Technology Committee (ARTC) issued a "Report on the Development of an Assisted Reproductive Technology System using Donor Sperm, Eggs and Embryos" [14] on April 28, 2003. This is an extremely important report because it sets out the conditions of eligibility for persons entitles to undergo ART treatment. The 6 main criteria are as follows:

(1) Persons who can receive reproductive medical treatment using donor sperm, eggs and embryos are limited to legal couples, excluding aged infertile couples (wife's age of 50 years is taken as a guide for an upper age limit).

(2) AID and IVF using donor sperm and eggs should be carried out only in the case of those couples who will remain infertile without being provided with sperm and eggs.

(3) Only embryos donated by another couple, who originally obtained embryos for their own use, can be used for embryo transfer, and embryos created by donor sperm and eggs may not be used.

(4) Cytoplasm or nuclear substitution of donor eggs is not allowed.

(5) Surrogacy is not allowed.

(6) Persons whose age is not less than 15 years can request the disclosure of information regarding their blood relationship on the basis of their right to know.

Further conditions regarding sperm and egg donors and other matters are as follows. With regard to (1), (2) and (3) above, donors of sperm should be male adults aged less than 55 years, and donors of eggs should be female adults aged not more than 35 years who already have a child. The number of children conceived using sperm or eggs from the same donor should be not more than 10. The donation should be carried out without charge other than actual costs, and should be made under a pseudonym. Donation from siblings is not allowed since anonymity is not guaranteed and human relationships in such a case are liable to become complicated. The retention period of sperm and eggs is 2 years, and that of embryos is 10 years. The donor sperm, eggs and embryos should be disposed of when the death of the donor is confirmed. The donor and donee should give informed consent after they have been fully informed about the procedures as well as the risks and benefits of ART, and opportunities for the withdrawal of consent and for counselling are assured. As reasons for (5), i.e. advocating a ban on surrogacy, various issues have been raised, and these are explored in detail under "surrogacy" in the Discussion section.

Further developments that should be noted are that on May 12, 2004, the Bioethics Specialized Investigation Committee in the General Council for Science and Technology announced that it would allow the production of fertilized eggs for ART studies only, and that it would permit IVF using sperm and eggs donated from third persons, the transfer of donor embryos, and subject to various conditions, the donation of sperm, eggs and embryos from siblings. Furthermore, the Cloning technology Control Law established in December 2000 regarded fertilized eggs as the germination of life, and allowed the production of fertilized eggs for research purposes on July 23, 2004. This question is the subject of ongoing debate in Japan.

It should be noted that all the reports and guidelines referred to here are advisory in nature and do not have the force of law.

#### ii) Deliberations on ART by other institutions

Compared with the ARTC report referred to under "governmental deliberations",, the Japan Society of Obstetrics and Gynecology adopts a strict attitude. The society announced on April 10, 2004 that it did not permit use of the fertilized eggs of a third person for fertilization since this would create complications in the parent-child relationship [15]. It approved the performance of pre-implantation genetic diagnosis for the first time on July 23, 2004, but this was a special arrangement for patients with muscular dystrophy [16]. Before this, a member who performed such a diagnosis without permission was expelled from the society, whereupon the member filed a complaint asking for the expulsion to be invalidated on the grounds that prohibition of pre-implantation genetic diagnosis violates the right of patients to pursue happiness as guaranteed under the Constitution of Japan.

In addition, the society does not approve of surrogacy [17] on the grounds that 1) the welfare of the resulting children should be assigned the highest priority, 2) surrogate conception carries physical risks and psychological burdens, 3) family relationships can become complicated, and 4) a surrogate conception contract is not ethically tolerated by society as a whole (is offensive to public order and morals). A member of the society was expelled because he facilitated the birth of a child by the use of a surrogate mother (the embryo of an elder sister and her husband was implanted in the womb of the younger sister, who acted as the surrogate mother). The society works through written notifications to disseminate its policies to its members. However, its opinions differ in detail from those of the government, and it is not in a position of being able to respond completely to rapid technological changes.

In addition to the above deliberations by the Japan Society for Obstetrics and Gynecology, there have been a number of separate initiatives by non-governmental bodies, all aimed at submitting drafts of, or making contributions to the development of, legislation on ART-related matters. These are listed below:

In June 1999, the National Institute for Research Advancement (NIRA) [18] launched a "Panel for Cloning and IVF" and prepared a Draft Law on the Morals of Life. The Draft Law would restrict cloning studies and ART, and prescribe the relationships between parents and children born by ART. It would allow the donation of eggs from an anonymous third person, prohibit the donation of embryos, prohibit donation between siblings, place a ban on surrogate conception or borrowed womb contracts, and restrict the age of donors [19,20].

In March 2000, the Japan Federation of Bar Associations provided advice and recommendations on ART through a document entitled "Legal Restrictions on the Usage of Reproductive Technology" [21]. It recommended, from the viewpoint of the prevention of ART abuse and the protection of the rights of users and resulting children, that a Reproductive Technology Law be formulated; the law should assure the right of children to know their blood relationship, place a ban on the donation of embryos, surrogate conception, or the use of a borrowed womb, and prescribe conditions limiting the use of ART and penalties.

In a separate initiative, the Japanese Society of Fertility and Sterility [22] submitted a written statement concerning the possibility of permitting IVF using eggs donated from siblings. It stated that it is necessary to harmonize the development of law and surrounding social circumstances (e.g., consideration to be given to whether or not to tell the truth to the resulting children, and discussions to be carried on between either party using IVF counsellors).

Finally, the Ethical Committee of the Japan Society of Fertilization and Implantation formulalted "Opinions and Advice concerning Assisted Reproductive Technology with a Donor's Semen" [23] to

It will be clear from the above that much of the groundwork for the drafting of a new law on ART-related matters has been done. Apart from the specialist view cited here, the other vitally important factor is the state of public opinion.

### Attitudes of citizens toward ART

**Figure 3:**
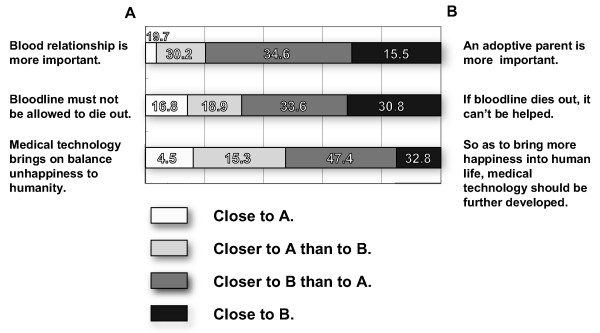
**National consciousness about ART-related medical technology and heredity**. This figure shows the attitude of Japanese citizens toward heredity. Results show that many feel the improvement of ART-related medical technology to be a necessary condition for the pursuit of happiness.

Mention was made at the beginning of this section of "Research on Attitudes of Citizens toward Assisted Reproductive Technology", issued as a report on a Scientific Special Research project carried out with the help of a Grant in Aid for Scientific Research from the Ministry of Health, Labour and Welfare in 2003. This report shows the attitudes of Japanese citizens toward ART. This research was conducted by using 8,000 male and female subjects aged 20 to 79 years [24]. The results of the research are tabulated in Figures 1, 2 and 3.

Figure 3 shows the attitude of Japanese citizens toward heredity. As mentioned in the "Background" section, the Japanese have traditionally shown strong feelings regarding the importance of "genetic blood relationship" and "continuation of the blood line". However, the results of research suggest that the importance attached to heredity has weakened, and that many people have come to think that caring for and bringing up a child is more important than the act of parturition. In addition, results showed that many feel the improvement of ART-related medical technology to be a necessary condition for the pursuit of happiness.

Figure 1 shows the penetration ratio of knowledge about ART in Japan. Approximately 50% of subjects knew about AID, IVF (donor sperms and donor eggs), and surrogacy. When the persons who answered "I've heard something about ART" are included, knowledge about ART has penetrated to approximately 90%. On the other hand, half of the subjects did not know about IVF (embryo transfer), and the penetration ratio of knowledge about IVF is low.

Figure 2 shows that when the question as to whether each type of ART should be socially accepted was asked, approximately 30% of subjects answered "I do not know", and many of them would not accept IVF using sperms and eggs donated from third persons, or surrogacy using eggs donated from third persons. Most subjects accepted other forms of ART, and especially, about 50% of the subjects would accept surrogacy using the fertilized egg of a couple who would like to receive ART.

**Figure 4:**
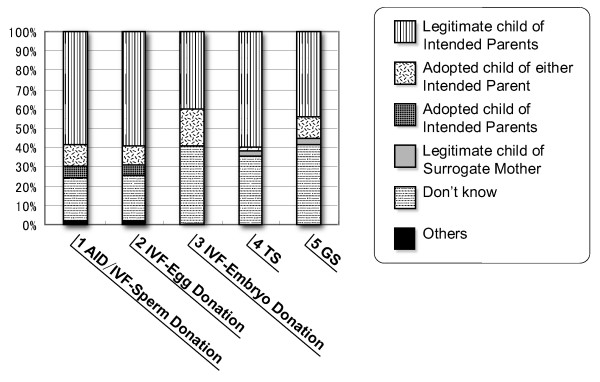
**National consciousness regarding parent-child relationships in cases of ART**. This figure shows national consciousness regarding parent-child relationships. Even embryos from third persons and surrogacy are accepted by approximately 40% of subjects.

In a separate research exercise, "Research on the Attitudes of Doctors and Citizens toward Assisted Reproductive Technology", carried out in 1998 [25], the relationship between children born by ART and their parents was investigated. Figure 4 shows the national consciousness regarding such parent-child relationships. Approximately 60% of subjects agreed that the children born by AID/IVF using sperms or eggs donated by third persons, by IVF using eggs from third persons, and by means of a borrowed womb, should be regarded as legitimate children of the concerned couples. Even embryos from third persons and surrogacy are accepted by approximately 40% of subjects. The subjects who answered "I do not know" were the second largest group. Approximately 10 % agreed that children born by ART should be regarded as adopted children of the concerned couples, and only 2% agreed that children born by borrowed womb should be regarded as adopted.

### Socio-ethical aspects of ART

Broadly speaking, it is possible to identify a wide variety of socio-ethical aspects of ART. In addition to surrogacy, these include social aspects, in particular the continuing social prejudice against ART still held in many quarters, the contradictions arising from the inherent failure of the law to catch up with technological development, economic aspects, in particular the financial burden on families, and the dilemma of how to reconcile the rights of children and the rights of parents. All these aspects or issues must be seen against the background of the continuing technological development of ART. A further underlying issue is the ethical one of how far science should be permitted to intervene in the field of human reproduction. These aspects are tabulated, for ease of reference, in Additional file 3, and are discussed, in terms of highlighting specific issues, in the following Discussion section.

## Discussion

### Court cases

So far, there have only been a small number of court cases concerning ART in Japan. In some cases, appeal proceedings are still continuing. Although much of the groundwork for comprehensive ART-related legislation has been laid, the legislation itself is still outstanding. The degree of understanding in society at large of the position of those couples that want to have children is also still insufficient. Despite these uncertainties, however, it is possible to categorize to some extent the criteria that have governed the thinking of the courts to date. These can be summed up as follows. a) The person who gives birth should be seen as the mother of the resulting child. b) It should be a condition that the husband of the concerned couple agrees to ART. c) Posthumous recognition can be accepted if there is a genetic relationship between the father and the child. d) The judgement should be made in the light of what is socially acceptable. In this section, we will examine each of these criteria in detail.

a) The person who gives birth should be seen as the mother of the resulting child. In other words, in determining legal parental status, primary importance is attached to the act of parturition. The underlying legal decision here is the 1962 decision of the Supreme Court, which ruled that "the parent-child relationship shall arise naturally based on the fact of childbearing without depending on recognition on the part of the mother" [26]. This thinking can be seen at work in court case iv) discussed under "Court Cases" above, when the Kobe Family Court ruled that the American woman who had given birth to twins should be seen as their legal mother. In this case, the egg used was donated, but even if it had come from the wife of the concerned couple, it is unlikely that the court's decision would have been different due to the importance attached to the 1962 Supreme Court precedent. As stated above, the decision of the Kobe Family Court was appealed to the Osaka High Court, and their decision was appealed to the Supreme Court, but in this case at least, there was no fundamental change in legal thinking. This same thinking also means that among the various patterns of ART, the only ones that raise problems regarding the determination of parental status are VII, when a borrowed womb is used, and VIII, i.e. surrogacy using the husband's sperm and donor eggs. The issue of surrogacy is further discussed later in this section.

b) It should be a condition that the husband of the concerned couple agrees to ART. In terms of court cases in Japan, this was a key issue in court case ii) concerned with denial of legitimacy, where the decision was influenced by the fact that the husband, Y, had not provided written consent to AID. On the basis of this kind of thinking and in that expressed in d), i.e. social acceptability, ART patterns II (AID), IV (IVF using donor sperm and eggs from the wife), V (IVF using sperm from the husband and donor eggs), and VI ((IVF using donor embryos), a potential source of problems is that in all these patterns there is a genetic links with a source extraneous to the concerned couple. With particular reference to pattern II (IVF), the main theories put forward about the legal status of resulting children are as follows: i) When the husband agrees to ART, the resulting child should be considered as a legitimate child in line with the legitimacy presumption of Article 772 of the Civil Code, ii) The resulting child should be considered to be a legitimate child that is not covered by the legitimacy presumption of Article 772 of the Civil Code since the husband has no blood relationship with the child even though he agreed to ART, and iii) This case is not covered by Article 772 of Civil Code, but if the husband is willing to adopt the resulting child, it can be adopted subject to the wife's agreement. A majority of people support theory i). However, the situation gets a little complicated when both husband and wife claim custody at a time of divorce. Scholars differ on what criteria should be given precedence when custody claims are settled. Many judicial precedents follow the principle of mother priority and allow the mother to have custody when the child is young. In addition, the courts display the line of thinking that the "rights of the father should be decided not based on biological or genetic viewpoints, but on social acceptance". If the husband consents and the ART performed is socially acceptable, the resulting child will be seen as the child of the husband. The Matsuyama District Court (court case iii) stated that "legally, we cannot completely reject ART that has been developed in response to the requests of couples that desire to have children" and "each case should be individually decided based on social acceptance until the enactment of legislation [27].

c) Posthumous recognition can be accepted if there is a genetic relationship between the father and the child. As referred to above under court case iii), the consent of the father and the existence of a blood relationship between the father and the child were considered sufficient by Takamatsu High Court in 2004 to override the time limit specified in the Civil Code. This decision is in itself important because traditionally posthumous recognition has been denied in Japan. However, this case has been appealed to the Supreme Court, and their decision, when handed down, will constitute a very important judicial precedent. There are several opinions concerning the court thinking as expressed in the above-mentioned court decision. Some people have no negative opinions about the claim for posthumous recognition because "there is a genetic relationship between father and child" and "the welfare of the child should be respected". These people are further divided into two groups: one group "approves posthumous recognition without reserve when there is a blood relationship", and the other "approves it while stipulating the father's agreement as a requirement". Other people have negative opinions about the claim for recognition because "the legal relationships will become complicated" and "this relationship goes beyond the legal parent-child relationship regulated by Civil Law". A similar trial was the Woodward case in the US [28]. In this case too, posthumous recognition was accepted since "there was a genetic relationship" and the "husband's agreement could be confirmed". The late husband was named as the father in the birth certificate of the resulting child and entitlement to a survivor's pension was permitted. The Japanese Ministry of Health, Labour and Welfare prescribes that sperm can only be stored for 2 years and should be disposed of when the death of the donor is confirmed, and does not recognize posthumous reproduction [29]. From the viewpoint of welfare of children, it might be preferable for the children who have a genetic relationship with their father and mother to be regarded as their legitimate children. As further technological advance are made, it seems almost certain that more people will wish to put their sperm and eggs into storage simply in order to make the bearing and upbringing of children fit with their chosen lifestyle. This in turn is likely to result in a plethora of complex legal problems. The situation merits careful observation in the future.

d) The judgment should be made in the light of what is socially acceptable. In terms of the cases discussed in this paper, this criterion is particularly relevant to case iv), the surrogacy trial. In this case, the Kobe Family Court followed the 1962 Supreme Court precedent, arguably very outdated since the judgement was handed down long before the days of ART. Much more important than this judgement is the still outstanding judgment of the Osaka High Court, and the case could well go as far as the Supreme Court. A Supreme Court judgement would have very wide legal and social implications, and in view of this, it is worth looking a little more closely, in this section at the legal issues, and in the next section at the socio-ethical issues. Of the three patterns of ART involving surrogacy, namely VII (borrowed womb using the embryo of a concerned couple), VIII (surrogacy using the husband's sperm and donor eggs), and IX (surrogacy using donor sperm and donor eggs), it has already been pointed out that in the case of IX, the necessity for a surrogacy contract is very questionable and there would seem to be no reason why a child born in this case should not be the subject of a standard general adoption procedure. In the case of patterns VII and VIII, however, the fundamental legal dilemma and the fundamental socio-ethical dilemma is how much importance to attach to a genetic link and how much to the act of parturition.

Looking at the legal issues related to surrogacy in a little more detail, it seems very unlikely that without new legislation, there will be no major change in Japan. In other words, as long as the courts continue to look at the 1962 Supreme Court judgment as the main precedent, overwhelming weight will continue to be put on the concept of parturition in terms of establishing the legal identity of the mother. Mention should also be made again here of the family register system in Japan, referred to under "Background" at the beginning of this paper. From the point of view of the welfare of resulting children, in view of social prejudices, it is highly desirable that children can be seen, in terms of the entry in the register, as the legally legitimate children of the concerned couple.

### Socio-Ethical Issues related to ART

#### Background trends

The issues discussed below should all be seen against a background of continuing technological development in various techniques related to ART. For example, it is expected that success rates in freezing eggs and sperm for later usage will improve rapidly, and as success rates in a particular technique improve, it is likely that more people will want to use it. Or for example, Intracytoplasmic Sperm Injection (ICSI) is proving effective in cases of severe infertility, though there are still concerns about the possibility of passing on genetic defects. In addition, cloning technology [30] can also be used in ART, but there are still unexplained aspects as well as technological problems in moving to actual applications. Alongside research into the technology of ART itself, research is also needed into what kinds of regulatory systems will need to be set up. Against this background, the following paragraphs will aim to give an overview of the main social-ethical issues related to ART insofar as these constitute the background against which court decisions are made and will be made in the future. The issue of surrogacy is again taken up as one such issue, this time from a socio-ethical perspective.

##### i) Surrogacy

Looking at the history of surrogacy in Japan, there have been 2 cases, in both of which relatives of the concerned couple became surrogate mothers by using the fertilized eggs of infertile couples, the first in May 2001 [31], and the second in March 2003 [32]. The arguments for and against surrogacy have continued until the present time. Arguments against have included a) increased risks, b) danger of custody battles, c) complication of family relationships, d) fear of commercialization, e) lack of a social consensus, and f) the usage of humans as a tool. The arguments have all been countered; it has been shown, for example, that the risks of childbearing are no different for a surrogate mother than for a general mother [33], and that there is also no difference in the risks for a child [34], or on the custody issue, that even in the US, there were custody battles in 2000 in only 8 out of 20,000 cases (0.04%) [35]. In terms of the relationship to the demographic situation, the number of children born through surrogacy outside Japan is increasing. Statistical data of accumulated ART cases [36] shows that one out of every 100 babies is born by IVF. This suggests that ART has potential as a measure to counteract the decrease in the number of children. At the present time, however, the government and the Japan Society of Obstetrics and Gynaecology are opposed to surrogacy, although a number of members of the Japan Society of Obstetrics and Gynaecology have said that "surrogate pregnancy is not acceptable, but it should be allowed under certain conditions if it is the only option". They have also gone to state very carefully that "In future, surrogacy will be accepted by more people as socially-accepted ideas change. If a social consensus to accept surrogate pregnancy were to obtain, surrogacy would come under review again in exceptional cases" [37].

On the other side of the fence, people argue for surrogacy citing g) the right to have children, h) reinforcement of traditional family values, i) children's welfare, and j) as a measure to counteract the decrease in the number of children. The "right to have children" can be derived from "the right to pursue happiness", guaranteed in Article 13 of the Japanese Constitution, and support for surrogate mothers is derived from "freedom of thought and conscience", derived from Article 19 [38].

We have looked in general terms at arguments for and against surrogacy, but it is worth looking briefly at the views of those with personal experience before delving more deeply into the opinions of people in general. One of the arguments against surrogacy mentioned above was the fear that people were being used as a tool. In fact, a surrogate-motherhood contract is not just a means of reproduction. A concerned couple and a surrogate mother communicate with each other for at least 10 months during pregnancy, and the surrogate mother does not give birth just as if she is carrying out a job without knowing the faces of her counterparts. In many cases, surrogate mothers felt that they carried out an act of friendship for infertile couples, and thought they had a good experience [39].

Reference has been made above and in other sections in this paper to the need for a social consensus. At least in Japan, even if there is not a total consensus, a sine qua non for any new legislation of the kind proposed is that a majority of citizens support it. According to research on citizens' attitudes in Japan, approximately 50% accepted the concept of a borrowed womb (Figure 2). In addition, a large number of citizens would put weight on the importance of childrearing rather than on the act of parturition. With particular reference to Pattern VII) ("borrowed womb"), it seems likely that it may not be too long before people come to accept this. One factor that should be mentioned in the context of the ongoing debate is that the number of abused children is increasing in Japan; this provides evidence for the view that the fact that a mother gives birth to a child is not by any means necessarily a guarantee that she will give that child a good upbringing. Indeed, it can and has been argued that the couples whose desire to have children is so great that they will even go as far as to use the procedure of surrogate mothers are likely to nurture the resulting children with affection.

##### ii) Social prejudice

Although, as has been mentioned above, about half the population are willing to accept the "borrowed womb" pattern of surrogacy, there is still considerable prejudice in Japan against ART. According to a questionnaire survey carried out by a publishing company with 3,433 female subjects receiving infertility treatment [40], approximately 50% had a job at the time of the survey, and about 39% had experienced a need to change or quit their job because of the treatment. In addition, they experienced not only physical, but also psychological pain caused by responses or words from the people around them, due to their infertility. No psychological or social support whatever is currently provided to either party receiving ART. As and when any legislation is passed, a system of counselling will need to be built into the resulting structure.

##### iii) Need for urgency and flexibility in legislation

When we focus primarily on the welfare of resulting children, it is clear that prompt improvement of legislation is required. However, since the law is unable to catch up with the development of scientific technology, inconsistencies will arise in many cases. It is therefore important to create a system in which responses can be promptly deliberated and implemented in such cases. In the US, the Uniform Parentage Act 2000 (UPA2000) [41] contains sections detailing the position of children born with the help of ART and that of gestational mothers, the term used in the UPA in place of surrogate mothers. Although UPA is currently adopted only in a minority of states, there is a likelihood that it could serve as one guideline for Japanese legislators in the future.

##### iv) Financial burden of ART

In Japan, the cost of health insurance is a charge on individual incomes. At a cost of about 30% of the actual expense, the people are able to use medical institutions. However, ART is not covered by the insurance, and the patients should bear 100% of the costs. However, from April 2004, each public body has given a subsidy of 100,000 yen (approximately 870 US dollars) for one course of treatment of ART, the subsidy to be granted (in the case of Metropolitan Tokyo) for not more than two courses of treatment. The amount of the subsidy is small, but the granting of a subsidy in itself indicates a step forward. However, the major burden still rests on the patients who are continuing to receive ART, and some people are forced to abandon it for financial reasons. This could well lead to a situation which high-income earners can have children by ART and low-income earners cannot.

##### v) The rights of children and the rights of parents

There is the possibility of conflict between on the one hand the right of self-determination on the part of parents who want to have children (Articles 11 and 13 of the Japanese Constitution) and on the other hand, the right of children to know details of their parentage [42]. In the case of children born with the help of ART, it is necessary to consider both children's "right to know" and their "right not to know". In the case of such children, giving them the right to know details of their parentage means that the secrecy and anonymity of those involved in the ART process (donors of sperm and eggs) is no longer protected. This may in turn produce a decline in the number of donors, resulting in a negative situation for infertile couples. And as far as parents are concerned, there will be a tendency to hesitate about giving children the "right to know". It can be argued that fundamentally, rather than prioritizing children's "right to know", what should be prioritized are the rights of parents who want to have children [43].

##### vi) An ethical issue – where to draw the line

A major issue in the case of ART is deciding how far science and technology should be permitted to intervene in the act of human reproduction. Within the field of natural phenomena, human reproduction is an act that has been constantly repeated. ART denotes the forcible application of science to this act. Furthermore, implicit in the act of donating sperm or offering the use of a womb is the possibility that "human beings" are being used as "things". On the other hand, however, there is the possibility of continuing change in terms of such issues as the development of ART technology or people's thinking about its ethical applicability [44]. In Japan at the present time, the population is declining. The number of infertile couples is also increasing. It is a fact that if we are to "maintain our descendants", there are times when it is necessary to call on the strengths of science for help.

## Conclusion

It will have become clear to readers of this paper that in terms of the central issue with which this paper is concerned, namely the determination of parental status in the case of children born with the help of ART, the Japanese courts still regard the act of parturition as the primary criterion for determining the legal mother. That said, the number of court cases involving ART in Japan is still small, but against the background of an increase in the number of infertile couples on the one hand and rapid development of ART technology on the other, it seems almost inevitable that difficult socio-ethical and legal problems will proliferate. A crucial factor in the ongoing debate is likely to remain that of the state of public opinion. The chief professional body concerned with ART, the Japan Society of Obstetrics and Gynaecology, has said with regard to surrogacy that: "In future, surrogacy will be accepted by more people as socially accepted ideas change. If a social consensus to accept surrogate pregnancy were to obtain, surrogacy would come under review again in exceptional cases." In relation to IVF, the courts have said that: "each case should be individually decided based on social acceptance until the enactment of legislation." Both of these statements support the author's view expressed above concerning the crucial nature of public opinion in relation to the acceptability of ART. At present, a substantial number of people have expressed a willingness to accept at least certain forms of ART, though there is still a wide divergence of opinion on the importance of maintaining the bloodline. Prior to the enactment of comprehensive legislation, a wide-ranging public debate is now needed. It is to be hoped that legislation will take into account the wide range of technical, moral and socio-ethical issues referred to in this paper, and as well as being comprehensive, will be flexible enough to respond rapidly to future changes.

The author intends to continue to research changes in the direction of court judgements on ART, changes in the consciousness of the people toward ART, and future deliberations by government and non-governmental bodies.

## Abbreviations

Assisted reproduction technology (ART), In vitro fertilization (IVF), Artificial insemination with donor's semen (AID), Artificial insemination with husband's semen (AIH), Intended Parents (IP), Gestational Surrogacy (GS), Traditional Surrogacy (TS)

## Competing interests

The author(s) declare that they have no competing interests.

## Authors' contributions

The author(s) declare that they have no competing interests.

## Pre-publication history

The pre-publication history for this paper can be accessed here:



## Supplementary Material

Additional File 1 **Table 1 – Judicial cases concerned with ART in Japan**. The number of court cases concerning ART is small in Japan. In some cases, the trial is still continuing, thus rendering the legal status of the resulting child unstable. Click here for file

Additional File 2 **Table 2 – AIH/AID/IVF/Surrogate mother**. This table shows the 9 main patterns of ART. Click here for file

Additional File 3 **Table 3 – Aspects of ART-related problems**. This table shows 6 background issues related to the technological and socio-ethical development of ART-related problems. Click here for file
