# Supplementary material for: Present state of reproductive medicine in Japan – ethical issues with a focus on those seen in court cases
Source: BMC Med Ethics. 2006 Apr 5;7:3. doi: 10.1186/1472-6939-7-3 (PMC1481581; doi:10.1186/1472-6939-7-3)
Supplement: Additional File 1 — Table 1 – Judicial cases concerned with ART in Japan. The number of court cases concerning ART is small in Japan. In some cases, the trial is still continuing, thus rendering the legal status of the resulting child unstable. [file 1472-6939-7-3-S1.pdf]

Table 1 Judicial cases concerned with ART in Japan

Case	AID	AID	AID	IVF	IVF	Surrogate mother
Date	30-Mar-98	16-Sep-98	18-Dec-98	12-Nov-03	16-Jul-04	14-Aug-04
Court	Niigata Family Court Nagaoka branch office	Tokyo High Court	Osaka District Court	Matsuyama District Court	Takamatsu High Court	Kobe Family Court Akashi branch office
Source	Family Court monthly report 1999, 51:179	The Law Times Report 2000, 1014:245	Hannrei Jihou 2000, 1696:118	The Law Times Report 2004, 1144:133	2003(Ne)497	
Claim	Appointment of person in parental authority	Appointment of person in parental authority	AID Denial of legitimacy	IVF Postmortem perception claim	IVF Postmortem perception claim	Claim for acknowledgement of legitimacy
Assertions by concerned parties	(Y)Legitimate child presumption extends to AID. (X)There is no blood relationship with the father.	(Y)Legitimate child presumption extends to AID. (X)There is no blood relationship with father.	(Y)The child was born from an adulterous act. (X)There was prior agreement by the husband, making the child legitimate.	Claim against the state for perception of legitimacy of a child born posthumously to the husband as a result of cryopreservation of husband's	Claim against the state for perception of legitimacy of a child born posthumously to the husband as a result of cryopreservation of husband's spermatozoon.	It is unreasonable for a birth certificate issued in the U.S.A. not be accepted in Japan.
Disposition	Husband's claim admitted	Wife's claim admitted	Husband's claim admitted	Claim dismissed	Claim admitted.	Claim dismissed
Procedural Posture	Agreement by both parties	The lack of a natural blood relationship has the possibility of influencing the child. It is appropriate for the mother to assume parental responsibility from	There is no proof of prior agreement or perception of a legitimate child by the husband.	Rather than genetic relationship, social acceptance is the determining factor in naming the father. In this case, social recognition was seen as insufficient.	There is a natural blood relationship and given the agreement of father, his physiucal presence is not a condition for peception of him as the father.	The woman who gave birth to the child should be seen as the mother and the matter should be settled by adoption.
