# Supplementary material for: Present state of reproductive medicine in Japan – ethical issues with a focus on those seen in court cases
Source: BMC Med Ethics. 2006 Apr 5;7:3. doi: 10.1186/1472-6939-7-3 (PMC1481581; doi:10.1186/1472-6939-7-3)
Supplement: Additional File 2 — Table 2 – AIH/AID/IVF/Surrogate mother. This table shows the 9 main patterns of ART. [file 1472-6939-7-3-S2.pdf]

Table 2 AIH/AID/IVF/Surrogate mother

	ART	Spermatozoon	Ovum	Pregnancy
I	AIH	Husband	Wife	Wife
II	AID	Donor	Wife	Wife
III	IVF	Husband	Wife	Wife
IV	IVF	Donor	Wife	Wife
V	IVF	Husband	Donor	Wife
VI	IVF	Donor	Donor	Wife
VII	GS	Husband	Wife	Donor
VIII	TS	Husband	Donor	Donor
IX	TS	Donor	Donor	Donor
