# Supplementary material for: Present state of reproductive medicine in Japan – ethical issues with a focus on those seen in court cases
Source: BMC Med Ethics. 2006 Apr 5;7:3. doi: 10.1186/1472-6939-7-3 (PMC1481581; doi:10.1186/1472-6939-7-3)
Supplement: Additional File 3 — Table 3 – Aspects of ART-related problems. This table shows 6 background issues related to the technological and socio-ethical development of ART-related problems. [file 1472-6939-7-3-S3.pdf]

Table 3 Aspects of ART-related problems

i	**Technical aspect**	Continuing technological development of ART may result in as yet inadequately researched side-effects and unforeseen social problems.
ii	**Social aspect**	Social prejudice against infertility and ART highlights need for support system. Volatility of public opinion.
iii	**Legal aspect**	Need for prompt and comprehensive legislation that can be adapted to meet technical advances.
iv	**Economic aspect**	Inequality and inadequacy of financial support puts additional financial burden on less well-off families.
v	**Family aspect**	Possibility of conflict between the rights of children and the rights of parents.
vi	**Ethical aspect**	Problem of where to draw the line in terms of the intervention of science into the act of human reproduction.
